# Fracture Resistance of Class II MOD Cavities Restored by Direct and Indirect Techniques and Different Materials Combination

**DOI:** 10.3390/polym15163413

**Published:** 2023-08-15

**Authors:** Vasiliki Tsertsidou, Petros Mourouzis, Dimitrios Dionysopoulos, Panagiotis Pandoleon, Kosmas Tolidis

**Affiliations:** 1 Department of Dental Tissues Pathology and Therapeutics, Division of Operative Dentistry, Faculty of Dentistry, Aristotle University of Thessaloniki, 54124 Thessaloniki, Greece; vtsertsi@gmail.com (V.T.); ddionys@dent.auth.gr (D.D.); ktolidis@dent.auth.gr (K.T.); 2 Department of Prosthodontics, Faculty of Dentistry, School of Health Sciences, Aristotle University of Thessaloniki, 54124 Thessaloniki, Greece; ppandoleon@dent.auth.gr

**Keywords:** short fibers, CAD/CAM, composite resin, crack propensity

## Abstract

This study aimed to evaluate the fracture resistance of class II MOD cavities restored using different techniques and materials. Sixty extracted maxillary molars were selected and standardized class II MOD cavities were prepared using a custom-made paralleling device. The specimens were divided into four groups based on the restoration technique used: Group 1 (direct resin composite), Group 2 (short-fiber-reinforced composite resin), Group 3 (composite polyethylene fiber reinforcement), and Group 4 (CAD/CAM resin inlays). Fracture resistance was assessed for each group after thermocycling aging for 10,000 cycles. The mode of fracture was assigned to five types using Burke’s classification. To compare the fracture force among the tested materials, a paired sample *t*-test was performed. The significance level for each test was set at *p* < 0.05. Significant differences in fracture resistance were observed among the different restoration techniques. CAD/CAM inlays (2166 ± 615 N), short-fiber-reinforced composite resin (2471 ± 761 N), and composite polyethylene fiber reinforcement (1923 ± 492 N) showed superior fracture resistance compared to the group restored with direct resin composite (1242 ± 436 N). The conventional resin composite group exhibited the lowest mean fracture resistance. The choice of restoration material plays a critical role in the clinical survival of large MOD cavities. CAD/CAM inlays and fiber-reinforced composites offer improved fracture resistance, which is essential for long-term success in extensive restorations.

## 1. Introduction

The search for the ideal restoration for severely compromised posterior teeth has been a significant challenge in restorative dentistry. Dental clinicians have documented, investigated, and explored various techniques and materials in their quest. Over one hundred published studies exist, focusing on the clinical performance of dental composites, which has created a competitive landscape within the field [1]. Such techniques encompass direct, semi-direct, and indirect approaches, [2] but the extensive range of available restorative materials often leads to confusion and misinterpretation among dental clinicians [3,4].

The loss of the marginal ridge, whether resulting from caries, endodontic treatment, or the removal of old amalgam restorations, has been identified as a critical factor affecting the survival of teeth [5]. Fatigue resistance is a significant property of new materials and a focal point of interest in novel restorative techniques. This is because the cumulative damage caused by cyclic loading and the presence of inherent flaws in a new restorative approach can lead to premature failure of the restoration [1,6].

Resin composite direct restorations constitute the primary modality in restorative dentistry, which are supported by well-documented studies investigating the mechanical properties [7,8], chemical properties [9] and biological properties of this material [10]. Nevertheless, the placement of large direct resin composite restorations presents challenges in terms of technique sensitivity and is associated with drawbacks such as dentin sensitivity [11] and potential microcracks in the enamel at the cusp base [12]. These complications are primarily attributed to polymerization shrinkage, which, in conjunction with contraction stresses, can give rise to marginal gaps and increase the risk of secondary caries [13]. Moreover, several studies have extensively examined adhesive posterior resin composite restorations using in vitro methods [14] and in silico simulations [15,16], confirming the significant impact of polymerization shrinkage, cusps deflection occlusal loading, enamel crack propagation, and cavity design on stress distribution. Understanding the redistribution of stresses in posterior restored teeth is crucial for preventing post-restorative problems. Notably, Class I and Class II posterior cavities, which were adhesively restored using a combination of shrinking filling materials, exhibited the most unfavorable stress concentrations in the replaced dentin and enamel tissues [17]. In the investigation of the fatigue strength of dental restorations, several methods have been utilized. These methods encompass in silico analysis [17], as well as in vitro experimentation [18]. Concurrently, clinical studies are focused on investigating the survival and success of diverse techniques utilized in the restoration of extensive cavities using polymer materials [19] or ceramic materials [20].

Finite element analysis (FEA) offers numerous advantages, as it allows for the analysis of intricate settings and provides detailed insights into the internal stress of teeth, restorations, materials, reduced time and cost to bring a new idea from concept to clinical application and the increased confidence in the final study [17,21]. However, FEA does have limitations concerning the assumption of isotropic elastic mechanical material behavior. This limitation arises from the fact that the materials under analysis are assumed to be isotropic and exhibit linear elastic behavior [22]. The most notable limitation of finite element analysis (FEA) is its heavy reliance on the model and assumptions made during the analysis [23]. The type, arrangement, and total number of elements employed in the model also influence the reliability of FEA results. Additionally, a significant drawback in many FEA studies within the field of dentistry is the incorporation of numerous assumptions concerning structural geometry, material properties, loading forces, bonding quality, and boundary conditions [24]. These assumptions can introduce uncertainties and may affect the accuracy and applicability of the FEA outcomes in practical clinical scenarios. As a result, traditional FEA with these assumptions may lead to less precise predictions and may not adequately capture the complex material response, especially when dealing with materials that demonstrate significant anisotropy or nonlinear behavior. On the contrary, in vitro studies face challenges in establishing a direct correlation between the number of cycles and load applied in the laboratory setting with the actual in vivo service time and masticatory activity. The primary aim of in vitro investigations is to replicate controlled, prospective, and longitudinal clinical scenarios, wherein restorations are placed under ideal conditions. However, it is crucial to recognize that the load to failure forces utilized in laboratory studies may not accurately represent those experienced in the oral environment. Consequently, failures observed in the in vitro setting tend to be more severe and less amenable to repair compared to real clinical situations. Therefore, caution must be exercised when extrapolating findings from in vitro studies to the clinical scenario [25,26].

Clinical assessment stands as the ultimate means to gauge the effectiveness of restorative materials and techniques. However, an array of influential variables, encompassing patients’ dietary habits, masticatory patterns, individual susceptibility to caries, and the involvement of multiple evaluators and operators, can attenuate the significance of the data, particularly within cross-sectional clinical investigations. Laboratory assessments exhibit limitations in capturing the authentic stress levels within the tested specimens, let alone pinpointing precise failure locations or mechanisms [27]. To address the issue of polymerization shrinkage, various concepts and materials have been introduced to the dental community. One such approach is the utilization of incremental layering techniques, which have been widely adopted by dental clinicians [28,29] and it has been established as the gold standard for the placement of resin composite [30]. However, alternative approaches such as the use of glass ionomer bases, [30], the incorporation of reinforcing fibers in resin composite [31], or the implementation of different polymerization protocols with bulk-fill resin composite have emerged as potential solutions to address the challenges posed by polymerization shrinkage [32,33]. Nonetheless, with the technological advancements in the field of dentistry, CAD/CAM restorations have emerged as a viable option. This technology enables dental clinicians to fabricate indirect restorations such as inlays, onlays, or crowns in a single visit. One of the advantages of CAD/CAM technology is the ability to control polymerization shrinkage, particularly in the case of large class II MOD cavities [34], because it confines it in the luting cement [34].

In recent times, short-fiber-reinforced composites (SFRC) have gained popularity for various restorative applications [35]. These materials hold great promise for crack arresting within their structure, which is attributed to unique features like aspect ratio, critical fiber length, fiber loading, fiber orientation, and matrix–fiber adhesion [36]. Notably, SFRC has demonstrated enhanced performance in both shallow and deep mesial–occlusal–distal (MOD) cavities concerning fracture resistance and fracture pattern [37]. In the realm of reinforcing composite restorations in high-stress-bearing areas, particularly in posterior teeth, SFRC has been recommended [38]. Studies have indicated that oblique layering of SFRC yields optimal results, showcasing increased fracture toughness comparable to natural dentition. This enhancement can be attributed to the low elastic modulus of the restorative material, which enhances its crack-blunting properties [38]. It is worth mentioning that incorporating fibers into dental resin composites has consistently shown superior mechanical performance when compared to non-fiber-containing resin composite restorative materials [39]. Furthermore, over the last two decades, a leno woven ultra-high molecular weight polyethylene fiber ribbon (Ribbond THM; Ribbond Inc., Seattle, WA, USA) has found application in various direct restorative techniques. The polyethylene fibers serve multiple purposes: firstly, to create a stress-absorbing layer and redirect potential cracks and fractures [40] and secondly, to internally splint the tooth and enhance fracture strength [41]. Application techniques involve either placing the fiber under the composite filling or applying the fibers circumferentially within the axial walls [41]. It is noteworthy that the randomly oriented fibers in short-fiber-reinforced composites (SFRC) provide reinforcement in three directions, whereas bidirectional or woven continuous fibers offer reinforcement in only two directions [42].

Nevertheless, theoretically, the directional distribution of reinforcement is less effective in SFRC, as a certain volume of fibers is divided into three directions [43]. To date, continuous bi-directional fiber-reinforced composites (EverStick NET; GC, Tokyo, Japan) have been used for reinforcing or repairing provisional fixed partial dentures or reinforcing indirect composite restorations by application in the intaglio [44] or placement under endocrowns [45], but not for direct restorative purposes inside cavities.

Within limits, there are two approaches to the usage of fibers in large class II MOD cavities [46]. The first approach involves the use of short-fiber-reinforced composite as a substructure within the dentin to reinforce composite restorations. The second approach utilizes braided long fibers made of polyethylene, which are inserted beneath composite restorations. The rationale behind these two approaches is that the polyethylene fiber network creates a distinct state of stress dynamics at the interface between enamel, composite, and adhesive, thereby enhancing fracture strength. This transfer of stress from the polymer matrix of the composite to the fibers contributes to improved mechanical properties [47].

The objective of this study is to evaluate the fatigue strength of class II MOD restorations by utilizing computer-aided design and computer-aided manufacturing (CAD/CAM) inlays as well as direct restorations employing resin composite, short-fiber-reinforced composite and composite polyethylene fiber. The null hypothesis (Ho1) states that there will be no significant difference in fracture resistance between class II MOD cavities restored using either restorative technique.

## 2. Materials and Methods

The study received approval from the Ethics Committee of Aristotle University of Thessaloniki (Approval No: 141/10-03-2022). The manufacturers, types, and compositions of the materials used in this study are listed in Table 1. A total of 60 caries-free mandibular molars without cracks were collected, cleaned, and scaled to remove plaque. These teeth were obtained from patients who provided their consent, including extracted molars due to periodontal reasons, from the clinics of undergraduate students at Aristotle University of Thessaloniki.

**Figure 1:**
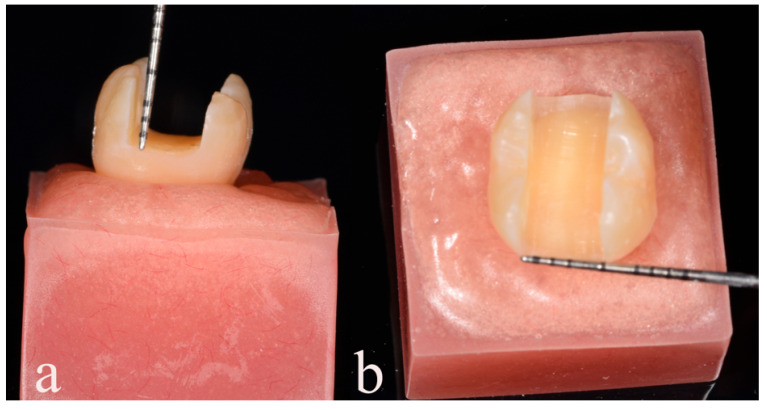
Standard MOD tooth preparation and corresponding measurements: (**a**) 5 mm in depth and (**b**) 5 mm in bucco-palatal width.

Prior to the cementation of all the restorations, a 1 mm thick layer of polyether material (Impregum, 3M ESPE, Seefeld Germany) was applied to the roots of each tooth to simulate the human periodontium. The teeth were then embedded in self-curing poly-methacrylate resin, ensuring that the crown and root ratio was maintained with the teeth inserted 1–2 mm below the cemento-enamel junction. Mesial–occlusal–distal type cavities were prepared on each tooth using a high-speed handpiece (Synea TK-98, W&H Dentalwerk GmbH, Bürmoos, Austria) and a specific diamond bur (Komet 845KRD025, Komet Trophagener Weg, Lemgo, Germany) with continuous water cooling. The cavity preparations were performed by an experienced clinician using a custom-made paralleling device to ensure standardized dimensions of 5 mm depth and 5 mm bucco-palatal width (Figure 1).

The 60 teeth were divided into four study groups based on the restorative technique and materials used. Group 1 (*n* = 15) was restored using a resin composite material (Tetric, Ivoclar Vivadent AG, Schaan, Liechtenstein) through a direct approach. After cavity preparation, a 37% phosphoric acid (Total Etch, Ivoclar Vivadent AG, Schaan, Liechtenstein) was applied, followed by the application of a self-bonding agent (Adhese Universal, Ivoclar Vivadent AG, Schaan, Liechtenstein) and layer-wise placement of a resin composite (Tetric, Ivoclar Vivadent AG, Schaan, Liechtenstein) with each layer being 2 mm thick. Each step was light cured for 20 s at 1500 mW/cm^2^ (Valo Curing Light, 505 West Ultradent Drive, South Jordan, UT, USA) according to the manufacturer’s instructions. Excess composite on the occlusal surface was then removed by polishing with a diamond bur.

In Group 2 (*n* = 15), short-fiber-reinforced composite (EverX posterior Bulk shade GC) was used in conjunction with resin composite following the manufacturer’s instructions. Initially, the internal surface of the cavity was etched with a 37% phosphoric acid gel for 30 s, and then a bonding agent (Adhese Universal, Ivoclar Vivadent AG, Schaan, Liechtenstein) was applied. The short-fiber-reinforced composite material was extruded from the dispensing tip and placed in a 4 mm layer close to the cavity walls. A plugger tip was used to adapt the material, and light curing was performed according to the manufacturer’s recommendations. The remaining cavity space was filled with resin composite. Finishing and polishing procedures were carried out using a polishing bur (H379AGK.314.023) to remove any excess material and achieve a clinical-like appearance.

In Group 3 (*n* = 15), a braided long fiber in the form of a polyethylene fiber (Ribbond) was inserted under the composite restoration (Tetric, Ivoclar Vivadent AG, Schaan, Liechtenstein). The Ribbond fibers were cut into pieces measuring 3 mm × 3 mm and 2 mm in thickness and were used as a reinforcement under the resin composite with 1 mm of thickness. Initially, the cavity was etched with a 37% phosphoric acid gel for 30 s, and a bonding adhesive (Adhese Universal, Ivoclar Vivadent AG, Schaan, Liechtenstein) was applied. The mesial, distal, internal, and occlusal walls of the cavity were built up with flowable composite (Tetric EvoFlow, Ivoclar Vivadent AG, Schaan, Liechtenstein). The pieces of Ribbond were wetted with the bonding adhesive and pressed through the composite against the interior tooth surfaces. Subsequently, the Ribbond pieces were cured, and the resin composite used for direct restorations (Tetric, Ivoclar Vivadent AG, Schaan, Liechtenstein) was placed over the Ribbond material incrementally in 1 mm layers, according to the manufacturer’s instructions. 

**Figure 2:**
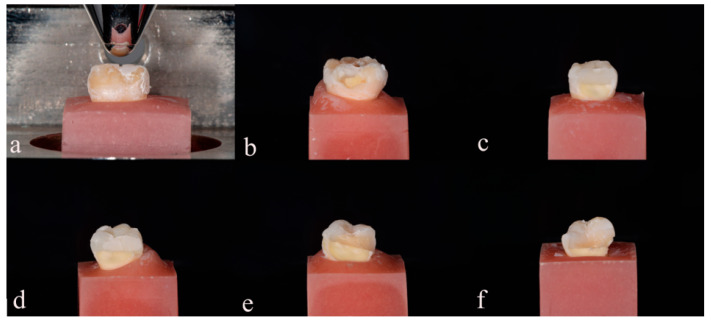
Side view of experimental setup. (**a**) Positioning of specimen for load-to-failure test. Fracture patterns of restored teeth: (**b**) Type I, minimal fracture in the crown; (**c**) Type II, less than half of the crown fracture; (**d**) Type III, half of the crown displaced; (**e**) Type IV, more than half of the crown lost; (**f**) Type V, severe fracture of the tooth and/or crown.

In Group 4 (*n* = 15), the CAD/CAM restorations were fabricated for each prepared tooth using a CAD/CAM approach. The teeth cavities were scanned with a CEREC Omnicam (Sirona Dental Systems GmbH, Bensheim, Germany). The design of the restorations was completed using CEREC 5.2 software, ensuring uniformity in form for all specimens. Brilliant Crios material was used for all the restorations in this group. The milling procedure was carried out using the fine mode and default milling burs (1.2 mm cylinder bur, Step bur). Following milling, the restorations were meticulously examined under a microscope (Zeiss, Pico) at ×16 magnification to check for any defects, cracks, or fit issues. After milling, the restorations were hand-polished according to the manufacturer’s instructions, using a clinical polishing set, resulting in a highly glossy restoration surface. The luting process for the restorations was performed in accordance with the manufacturer’s instructions. The inner surface of each restoration was etched with 4% hydrofluoric acid (IPS Ceramic, Ivoclar Vivadent AG, Schaan, Liechtenstein) for 60 s, followed by rinsing with water for 30 s and air drying for 10 s. Ceramic primer (Clearfil Ceramic Primer Plus, Kuraray Noritake, Hattersheim, Germany) and Panavia Tooth Primer were then applied as per the manufacturer’s instructions. The CAD/CAM restorations were finally cemented using Panavia V5 (Kuraray Noritake, Hattersheim, Germany). After the restorations were placed, all the teeth were stored in an incubator at 37 °C and 100% humidity for 24 h prior to thermocycling aging. All the tooth specimens were artificially aged using a thermocycling procedure of 10,000 cycles in deionized water solution at 5–55 °C, with a transfer time of 5 s and dwelling time of 30 s. In total, 10,000 cycles corresponded to 1 year of clinical use [48]. The teeth were subjected to a compressive load applied to the occlusal surface, using a servo hydraulic material test system (M350-10kN, Testometric AΧ, Rochdale, UK) at a crosshead speed of 0.5 mm/min. A round-ended steel cylinder with a radius of 1.3 mm (Figure 2) was used for the testing. The peak force required to fracture the tooth was measured and recorded in Newtons. Following the fracture resistance test, the restorations were promptly identified and classified into five distinct types utilizing the classification system outlined by Burke et al. [49]. This classification procedure was coupled with standardized photography. The specimens were then categorized according to the specific nature of crown failure, delineated as follows:

Type I—Minimal fracture or minor crack within the crown;
Type II—Loss of less than half of the crown structure;
Type III—Crown fracture extending through the midline, with displacement or loss of half of the crown;
Type IV—Loss of more than half of the crown structure;
Type V—Severe fracture involving both tooth and crown components.

### Statistical Analysis

The peak force values for fracture in all specimens were assessed for normality using the Shapiro–Wilk test, and homogeneity of variance was examined using Levene’s test. The test results demonstrated that the data met the assumptions of normality and homogeneity of variance, ensuring the robustness of the statistical analysis. To compare the fracture force among the tested materials, a paired sample *t*-test was conducted with meticulous care. The statistical analysis was performed using IBM Statistics software (version 29.0), following standard methodologies. A significance level of *p* < 0.05 was applied to determine statistical significance, indicating that results with a *p*-value below this threshold were considered statistically significant. By adhering to these specific and well-defined statistical procedures, the study aimed to ensure the reliability and accuracy of the statistical findings.

## 3. Results

**Figure 3:**
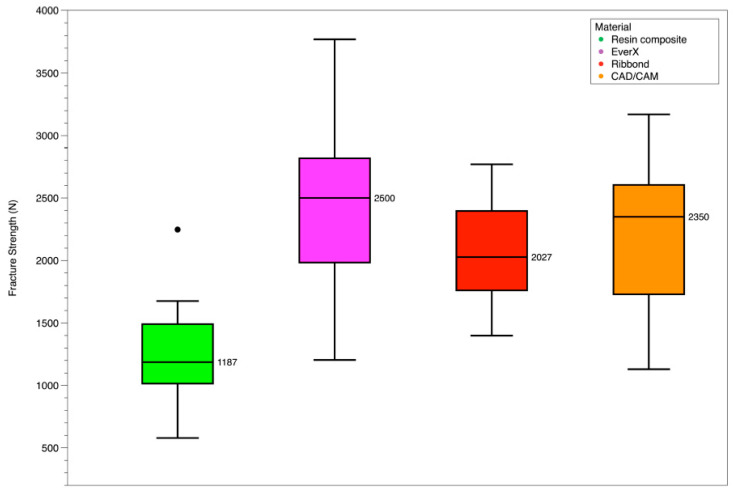
Box plot of the effect of material on fracture strength of restored MOD cavities in teeth.

The mean values and standard deviations of the peak fracture force (in Newtons) are provided in Table 2. Additionally, these values are visually represented in Figure 3 for a better understanding of the data. Table 3 provides the mode of fracture distribution of the four restorative groups according to Burke’s classification [49]. Illustrations of each type of fracture are shown in representative photos in Figure 2b–f.

### Peak Fracture Toughness

The present study observed the highest fracture force for the EverX group (2471 ± 761 N), whereas the lowest values were recorded for the resin composite group (1242 ± 436 N). Statistical analysis revealed significant differences between the resin composite group and the short-fiber-reinforced composite group (paired sample *t*-test, t(14) = −5.545, *p* < 0.001). Furthermore, comparisons between the resin composite group and the composite polyethylene fiber reinforcement group, as well as the CAD/CAM restorations group, also showed statistically significant differences (paired sample *t*-test, t(12) = −4.370, *p* < 0.001 and t(14) = −4.112, *p* = 0.001, respectively). However, no significant differences were found when comparing the CAD/CAM group with the short-fiber group and the composite polyethylene fiber group (paired sample *t*-test, t(14) = −1.264, *p* = 0.227 and t(10) = 0.691, *p* = 0.505, respectively). Additionally, the comparison between the short-fiber group and the composite polyethylene fiber group did not yield a statistically significant result (paired sample *t*-test, t(10) = 1.659, *p* = 0.064).

## 4. Discussion

This study aimed to compare the fracture resistance of class II MOD cavities restored using different techniques and materials. The results revealed a significant difference in fracture resistance among the restorative groups investigated. This finding led to the rejection of the null hypothesis, indicating that all three approaches (short-fiber-reinforced composite, composite polyethylene fiber reinforcement, and CAD/CAM) exhibited superior fracture resistance compared to the group restored with resin composite alone. The EverX group, which utilized short-fiber-reinforced composite, demonstrated the highest mean fracture resistance, whereas the conventional resin composite group showed the lowest mean fracture resistance.

It is worth noting that the posterior region of the oral cavity experiences significant forces during mastication, ranging from 300 to 600 Newtons [50]. Therefore, achieving optimal fracture resistance in restorations placed in this area is crucial for long-term success and the preservation of tooth structure. The findings of this study highlight the potential benefits of using alternative techniques and materials, such as short-fiber-reinforced composite and composite polyethylene fiber reinforcement, to enhance the fracture resistance of class II MOD restorations. Additionally, the CAD/CAM approach showed promising results, providing clinicians with a convenient and reliable method for indirect restorations in a single visit [51]. However, higher forces have been observed in cases of bruxism [52]. Furthermore, teeth situated in the posterior region can be exposed to remarkably high forces under specific circumstances, such as inadvertent biting on hard objects or experiencing trauma [50]. These forces have the potential to surpass those encountered during routine mastication or instances of bruxism [50]. Hence, it is imperative to underscore the exceptional demands placed on restorations in the posterior region, particularly concerning their fracture resistance. The intricacy of such restorations escalates significantly when dental clinicians are faced with extensive destruction of dental tissues in this anatomical area [18].

In the present study, conservative and minimally invasive treatment approaches were implemented to restore class II MOD cavities. Restorations were performed utilizing resin composite, short-fiber-reinforced composite, reinforcement with polyethylene fiber, and CAD/CAM inlays, following the protocols and instructions provided by the respective manufacturers. The principal aim of this investigation was to ascertain the optimal approach for managing class II MOD cavities, with a specific focus on evaluating their fracture resistance, which is a critical aspect in the context of these challenging dental restorations.

A static loading test was conducted to evaluate the performance of the restorations until failure occurred. Prior to the loading test, the restorations underwent artificial aging through thermocycling. This aging process was implemented to assess the durability and stability of the restorations under conditions that simulate the oral environment [48].

Subsequently, the restorations were subjected to a compressive force applied perpendicular to the occlusal surface of the teeth until failure was observed. In this study, the implementation of different techniques and materials underwent investigation of a load-to-failure fatigue test. Other methods of analysis such as in silico analysis offer advantages but are more focused on isotropic materials, and although clinical studies are more accurate, they need more time to complete and have the major disadvantage of different intraoral conditions due to different patients treated. Both clinical and experimental studies are confronted by an array of confounding factors, including the potential for observer or operator bias, variations in tooth anatomy, procedural flaws, equipment calibration, and other intricacies. Thus, meticulous consideration of these elements remains pivotal when interpreting the outcomes of such research endeavors [27].

To ensure tripod contact and replicate the natural occlusal forces experienced during mastication, the load was distributed onto the buccal and lingual cusps of the teeth. This was achieved by utilizing a 6 mm-diameter stainless-steel sphere as a contact point, allowing for the even distribution of forces across both the buccal and lingual cusps. By employing this methodology, realistic loading conditions for the tooth/restoration specimen during the fracture test were simulated [53].

The primary limitation of resin composite materials lies in their inherent polymerization shrinkage during the curing process. This shrinkage phenomenon can give rise to gaps between the composite and the tooth structure, consequently compromising the initial fracture resistance of the material. This effect becomes more pronounced in extensive restorations, as seen in our laboratory research, where resin composite materials exhibit significantly higher contraction stresses [26,54]. This concern can be somewhat mitigated through strategies such as incremental layering. An additional complication of composite restorations lies in their deficiency in terms of fracture toughness. Contemporary composites exhibit robust mechanical properties, yet they lack the desired fracture toughness necessary for countering crack propagation under loading conditions, a characteristic which is pivotal for addressing cavities in posterior teeth [55].

Recognizing this challenge, novel products have been developed to mitigate these limitations [56]. The utilization of fiber reinforcement in dentistry has been well-documented in the literature for several decades. Numerous studies and research articles have highlighted the benefits and effectiveness of incorporating fiber reinforcement in various dental applications [57].

These fibers have demonstrated their ability to enhance the mechanical properties and performance of restorative composite materials [58]. The incorporation of polyethylene fiber reinforcement into dental resins can enhance their mechanical properties and overall performance. Such fibers manifest the potential to fortify both the restoration itself and the structurally compromised tooth [47]. Notably, the dimensions, type, and orientation of these fibers can significantly influence the potential strengthening effect of these materials. In the case of short-fiber-reinforced composites (SFRC), the fibers exhibit random orientations, thereby contributing to reinforcement in multiple directions. These elongated fibers can serve as an intrinsic splint, effectively connecting the residual tooth structure [42]. These polyethylene fibers boast a dense concentration of fixed nodal intersections, which substantiates the fabric’s integrity. This structural feature facilitates the more efficient transmission of stresses throughout the material due to well-defined load pathways [59]. SFRCs offer practicality and efficiency in dentin replacement, though the random fiber orientations might not achieve the utmost reinforcement potential. Long fibers, when utilized to stabilize opposing walls, not only act as internal splints but also as prospective stress-absorbing layers. The favorable performance of polyethylene fibers arises from their distinctive properties, chemical bonding with resin, the influence of the leno weave in terms of crack resistance and deflection, and the capacity to resist shifting within the matrix [59]. An additional facet of SFRCs and polyethylene fibers is their role in potentially redirecting or halting crack propagation within restorations. This is crucial for achieving optimal fracture patterns, as the pattern significantly influences the restorability of teeth following fracture incidents. Fibers have demonstrated their capacity to redirect and/or arrest crack propagation within composite restorations [60], distinguishing them from composite materials with substantially lower fracture toughness relative to dentin, rendering them incapable of effectively halting crack propagation.

These fibers serve as load-bearing elements, effectively distributing stresses and preventing crack propagation [61]. Despite the promising results, the widespread adoption of fiber reinforcement in routine clinical practice has been limited due to various factors. These factors include the complexity of the technique, sensitivity to proper application, and the absence of robust evidence-based guidelines [18].

Although the use of fiber reinforcement has shown potential in improving the mechanical properties of dental materials, its integration into everyday practice requires careful consideration and further research [62]. In this study, the investigated group demonstrated satisfactory outcomes, offering advantages such as increased strength, toughness, and fracture resistance to the restorations. In 2013, a new restorative composite called EverX Posterior was introduced to the market. This composite was specifically developed to address the challenges of durability in medium to large cavities, particularly in the posterior region of the oral cavity. Its primary objective was to provide enhanced resistance against cracking and fracture caused by excessive forces in this area. To evaluate its performance, both in vitro and clinical tests were conducted on EverX Posterior. These studies demonstrated that this composite exhibited superior mechanical properties and polymerization stress compared to conventional composites [63]. This finding is of significant importance as it suggests that EverX Posterior has the potential to serve as a biomimetic restorative material, closely mimicking the natural properties of teeth [31].

The limitations of this study provide valuable insights for a comprehensive interpretation. Firstly, the employed experimental approach involves the placement of a rigid sphere within excised teeth exhibiting MOD cavities. Although this approach simplifies the assessment of MOD strength, it is important to acknowledge that the complex interplay of lateral and rotational pressures on cusp surfaces, coupled with the intricate dynamics of mouth movement and occlusal interactions, extends beyond the scope of simple axial force application. Additionally, the use of freshly extracted intact teeth, while ensuring controlled conditions, contrasts with the dynamic nature of dental damage often encountered in clinical scenarios. Clinical practice involves cavity preparations with inclined surfaces, complex base configurations, and potential undercuts, which are tailored to the extent of carious lesions. It is noteworthy that the exclusive inclusion of caries-free mandibular molars was essential for standardizing preparatory procedures, aligning with practices followed by other researchers. Furthermore, the clinical dental environment introduces not only mechanical stresses but also metabolic changes that impact dental restorations. This interplay may influence the gradual degradation of the interfacial bond between the restoration and the tooth. To mimic clinical function, cyclic loading was implemented. Given these study limitations, a nuanced interpretation of the findings is recommended. Future directions should explore randomized clinical trials and further comparative studies that share the objectives of this investigation.

The findings of this study align with previous research in the field, supporting the notion that short-fiber-reinforced composites demonstrate enhanced fracture toughness, making them a favorable choice for high-stress bearing applications, especially in cases involving extensive damage to dental tissues. CAD/CAM inlays offer an alternative approach for restoring class II MOD cavities. This indirect restorative method has gained widespread acceptance and utilization in modern dentistry. CAD/CAM inlays provide several advantages over direct restorations, including improved fatigue resistance and a reduced risk of cracks in class II MOD restorations. A study conducted by Silva et al. found that large direct conventional composite restorations were significantly more prone to fracture due to polymerization shrinkage pressures compared to CAD/CAM composite inlays [26]. Furthermore, an important advantage of composite inlays is that the polymerization of the composite materials takes place before the clinical treatment thus mitigating the detrimental effects of polymerization shrinkage strains on the tooth structure. In their laboratory study, Papadopoulos et al. similarly highlighted that CAD/CAM inlays serve as an alternative restorative material, yielding satisfactory outcomes in terms of improving the prognosis of extensive MOD restorations, particularly in relation to their fracture resistance [53]. The results obtained in this study are consistent with the aforementioned research as the groups that received CAD/CAM inlays and fiber reinforcement demonstrated superior fracture resistance compared to the direct placement of resin composite.

## 5. Conclusions

In conclusion, considering the limitations of this study, it can be inferred that the utilization of CAD/CAM technology and fiber reinforcement techniques in the restoration of class II MOD cavities provides notable advancements in the long-term prognosis of extensive posterior tooth restorations, specifically in terms of fracture resistance, when compared to resin composite alone. Notably, the implementation of short-fiber-reinforced composite demonstrated the most favorable outcomes in terms of fracture resistance for teeth with large class II MOD cavities, surpassing other approaches examined in this study. However, it is important to emphasize that further investigation and clinical trials are warranted to establish more durable and effective clinical protocols for cases involving significant dental tissue destruction.

## Figures and Tables

**Table 1:** Description of materials according to the manufacturer.

Material	Type	Composition		Manufacture
**Ribbond**	Polyethylene fibers	Preimpregnated, silanized, plasma-treated, leno-woven, ultra-high modulus polyethylene fibers.		Ribbond
		**Matrix**	**Fillers**	
**EverX posterior**	Millimeter-scale short-fiber-reinforced composite	Bis-GMA, TEGDMA, PMMA.	Silicon dioxide (max. 5 wt%), Barium glass (max. 70 wt%) E-glass fiber (max. 15 wt%).	GC
**Brilliant Crios**	nano-hybrid CAD/CAM composite block	(28.4 wt%) cross-linked Bis-GMA, bis-EMA, UDMA	Amorphous SiO_2_ (<20 nm), barium glass (<1 nm), bis-EMA, UDMA, inorganic pigments: ferrous oxide or titanium dioxide	Coltene Whaledent AG
**Tetric**	Nano-hybrid composite	(18.8 wt%) BisGMA, TEGDMA, UDMA	Barium glass filler, Ytterbium trifluoride, mixed oxide (63.5 wt%), polymer (17 wt%), additive, catalysts, pigments, stabilizers (0.7 wt%) Particle size: 0.04–3 μm	Ivoclar Vivadent Schaan, Liechtenstein.

Bis-GMA = bisphenol A glycol dimethacrylate; Bis-EMA = ethoxylated bisphenol A dimethacrylate; TEGDMA = Triethylene glycol dimethacrylate; UDMA = Urethane dimethacrylate; PMMA = poly(methyl methacrylate).

**Table 2:** Mean fracture toughness and standard deviation.

Material	Peak Fracture (N)
Resin composite	1242 ± 436
EverX	2471 ± 761
CAD/CAM	2166 ± 615
Ribbond	1923 ± 492

**Table 3:** Mode of fracture distribution of the restorative groups according to Burke’s classification.

Mode of Fracture	Group 1 Resin Composite	Group 2 E verx	Group 3 Ribbond	Group 4 CAD/CAM
TYPE I (Minimal fracture or crack in the crown)	0	2	4	0
TYPE II (Less than half of the crown lost)	0	9	4	3
TYPE III (Half of the crown displaced or lost)	5	4	5	7
TYPE IV (More than half of the crown lost)	6	0	2	5
TYPE V (Severe fracture of the tooth and/or crown)	7	0	0	0

## Data Availability

Not Applicable.
